# Evaluation of the SH-SY5Y cell line as an *in vitro* model for potency testing of a neuropeptide-expressing AAV vector

**DOI:** 10.3389/fnmol.2023.1280556

**Published:** 2023-11-30

**Authors:** Jeanette Zanker, Daniela Hüser, Adrien Savy, Sara Lázaro-Petri, Eva-Maria Hammer, Christoph Schwarzer, Regine Heilbronn

**Affiliations:** ^1^Department of Neurology, AG Gene Therapy, Berlin Institute of Health, Charité—Universitätsmedizin Berlin, Corporate Member of Freie Universität Berlin, Humboldt-Universität zu Berlin, Berlin, Germany; ^2^Institute of Pharmacology, Medical University of Innsbruck, Innsbruck, Austria

**Keywords:** adeno-associated virus, gene therapy, neuropeptide, SH-SY5Y, differentiation

## Abstract

Viral vectors have become important tools for basic research and clinical gene therapy over the past years. However, *in vitro* testing of vector-derived transgene function can be challenging when specific post-translational modifications are needed for biological activity. Similarly, neuropeptide precursors need to be processed to yield mature neuropeptides. SH-SY5Y is a human neuroblastoma cell line commonly used due to its ability to differentiate into specific neuronal subtypes. In this study, we evaluate the suitability of SH-SY5Y cells in a potency assay for neuropeptide-expressing adeno-associated virus (AAV) vectors. We looked at the impact of neuronal differentiation and compared single-stranded (ss) AAV and self-complementary (sc) AAV transduction at increasing MOIs, RNA transcription kinetics, as well as protein expression and mature neuropeptide production. SH-SY5Y cells proved highly transducible with AAV1 already at low MOIs in the undifferentiated state and even better after neuronal differentiation. Readouts were GFP or neuropeptide mRNA expression. Production of mature neuropeptides was poor in undifferentiated cells. By contrast, differentiated cells produced and sequestered mature neuropeptides into the medium in a MOI-dependent manner.

## 1 Introduction

Since their discovery in the late 1960s (Atchison et al., 1965; Hoggan et al., 1966) and the subsequent cloning of the AAV genome into plasmids in the early 1980s (Samulski et al., 1982), recombinant AAV vectors (rAAVs) have been intensely studied and become an integral part human gene therapy. Upon infection, AAV capsids attach to cellular receptors, are internalized through endocytosis, and are transported through the Golgi apparatus. The capsids then escape the endosomes and are transported to the nucleus through the nuclear pore complex. AAV vectors have to reach the cell nucleus for the release of the DNA, complementary strand synthesis, and gene expression (Dhungel et al., 2021; Riyad and Weber, 2021). As a single-stranded DNA virus, AAV ssDNA genomes require double-strand DNA generation either by complementary-strand synthesis or by complementary-strand annealing (Ferrari et al., 1996; Nakai et al., 2000) before mRNA transcription can be initiated. The wide range of natural or engineered capsid serotypes allows targeting of distinct organs or cell types ranging from the liver, muscles, and eyes to the central nervous system (CNS) (Snyder et al., 1997; Lo et al., 1999; Nathwani et al., 2007). Moreover, rAAV has been shown to be safely delivered into the CNS, following direct intraparenchymal injection and systemic routes of administration (Haery et al., 2019).

With the emergence of rAAV applications *in vivo, in vitro* assay development for characterization of the vector functionality has been growing. While methods for physico-chemical AAV characterization have been established and with their robustness proven, the development of transgene-specific assays for the assessment of vector potency and their *in vitro* set-up can be challenging. In several instances, post-translational modifications are required to yield biologically active transgene products. This requires an understanding of the relevant biological mechanism of action. The potency assay should reflect this mechanism to assess the suitability of the vector for *in vivo* use in research and to fulfill the regulatory requirements for gene therapies as stated by the authorities in their guidelines (FDA, 2011; EMA, 2018). Therefore, the evaluation of an appropriate cellular model is the prerequisite to reduce the use of animal experiments as recommended by regulatory authorities (FDA, 2011; EMA, 2018), the rise of three R implementation (Neuhaus et al., 2022), as well as mitigations for the requirement of animal testing in the United States (Wadman, 2023).

In AAV gene transfer for neurological research and therapeutic applications, differentiated human SH-SY5Y cells have been shown to be permissive for different AAV serotypes such as AAV2 (Dudek et al., 2010; You et al., 2012; Lewis et al., 2015; Osborne et al., 2018), AAV5 (You et al., 2012), AAV1 (Patricio et al., 2018), and some hybrid capsids (Charbel Issa et al., 2013). They have been shown to express proneuropeptide Y from an AAV1 vector (Patricio et al., 2018) and antiapoptotic genes from the Bcl-2 family (Garrity-Moses et al., 2005; Kermer et al., 2015) in the context of Parkinson's disease research. This makes this cell line an appealing option for the development of an *in vitro* potency assay for neuropeptide-expressing AAV1 vectors. It has, however, not been shown so far whether SH-SY5Y cells are capable of processing precursors of neuropeptides. This step is essential to yield mature active peptides, ready for functional studies.

In the present study, we investigated differentiated and undifferentiated SH-SY5Y cells in parallel for transduction of a preprodynorphin-encoding rAAV1 for the production of fully processed dynorphin peptides by applying antibodies for specifically detecting mature dynorphin B but not its precursor fragments.

## 2 Materials and methods

### 2.1 Cell culture and SH SY5Y differentiation

Human embryonic kidney (HEK) 293 (ATCC—no. CRL-1573; RRID:CVCL_0045) cells were cultivated in Dulbecco's Modified Eagle's Medium (DMEM; Gibco—no. 41966-029) supplemented with 10% fetal bovine serum (FBS; Pan Biotech—no. P30-3031). The human neuroblastoma cell line SH-SY5Y (ECACC—no. 94030304; RRID:CVCL_0019) was cultivated in RPMI-1640 (Sigma-Aldrich—no. R8758) supplemented with 10% FBS. SH-SY5Y cell lines were reported to be cross-contaminated with mouse cells (Jiang and Wang, 2014). Cells obtained directly from ECACC as used in this study were found to be negative for mouse RNA by RT-ddPCR using mouse GAPDH targeting primers/probe (Supplementary Table 1).

For differentiation, cells were plated either at 4 × 10^4^ cells/well in a 12-well plate format (Falcon—no. 353043) or at 7 × 10^4^ cells/well in a six-well plate format (Falcon—no. 353046) (Shipley et al., 2016). SH-SY5Y cells were differentiated with RPMI-1640 medium supplemented with 2% FBS, 10 μM retinoic acid (RA; Sigma-Aldrich—no. P2625), and 16 nM 12-O-tetradecanoyl-phorbol-13 acetate (TPA; Sigma-Aldrich—no. P8139) 5 days before rAAV transduction (You et al., 2012; Charbel Issa et al., 2013; Mendsaikhan et al., 2018). Medium was renewed every 48 h. As a differentiation control, RMPI-1640 medium supplemented with 2% FBS and 0.2% DMSO (Santa Cruz Biotechnology—no. sc-358801) was used.

### 2.2 AAV constructs

Self-complementary (sc) AAV vector plasmids for the expression of codon-optimized versions of human preprodynorphin (pDyn) cDNA or cytoplasmic-enhanced green fluorescent protein (eGFP) were derived from previously characterized plasmids. The transgenes were expressed by the ubiquitous CBA promoter and terminated by the bovine growth hormone poly A signal sequence (Agostinho et al., 2019). Single-stranded (ss) AAV vector plasmids for pDyn and eGFP were constructed into the pTRUF11 backbone (Burger et al., 2004). The single-stranded constructs contained the woodchuck hepatitis virus posttranscriptional regulatory element (WPRE).

### 2.3 rAAV1 production

rAAV1 was produced in adherent HEK293 using a dual-plasmid transfection system as previously described (Grimm et al., 2003; Zanker et al., 2022). In brief, HEK293 was co-transfected with rAAV vector plasmid and pDP1rs (PlasmidFactory—no. PF401) with polyethylenimine (PolyScience—no. 23966-1). After 48 h of scAAV or 72 h of ssAAV transfection, the cells and the medium were harvested and purified with a Poros AAVX Capture-Select Affinity Chromatography Column (Thermo Fisher—no. A36652) over an ÄKTA purifier (Cytiva). rAAV titers were determined with ddPCR targeting the promoter region.

### 2.4 Evaluation of eGFP transduction efficiency

eGFP expression was assessed by flow cytometry following rAAV-eGFP transduction. Cells were harvested using TrypLE Express (Gibco—no. 12605010) and resuspended in 1 mL of Dulbecco's phosphate-buffered saline (DPBS; Thermo Scientific—no.14190-094) supplemented with 10% FBS. eGFP expression was assessed using the BD FACSCalibur Flow Cytometer (BD Biosciences). Samples were analyzed using the BD CellQuest Pro Software (BD Biosciences—Version 4.0.2).

### 2.5 Quantification of vector genome copy numbers by ddPCR

rAAV-eGFP vector genome copy numbers were assessed by ddPCR on cellular DNA. The cells were washed three times with DPBS before harvest with TrypLE Express. The cells were resuspended in 1 ml of DPBS supplemented with 0.05% Pluronic F-68 (Thermo Scientific—no. 24040032). Then, the cells were pelleted at 400 × *g* for 4 min and snap-frozen in liquid nitrogen. DNA was extracted from cell pellets by proteinase K (PK) (Roth—no. 7528.5) treatment followed by EtOH precipitation with sodium acetate (NaAc) (Sigma Aldrich—no. S2889-250G) as described before (Zanker et al., 2022). In brief, the cells were resuspended in 100 μl DPBS and treated with 1 mg/ml of PK at 72°C for 15 min. A 3 M NaAc was added at a 1:10 (v/v), followed by four volumes of absolute ethanol. Samples were stored for 30 min at −80°C. DNA was pelleted by centrifugation at 17,000 × *g* for 15 min at 4°C, followed by two wash steps using 70% ethanol. DNA was resuspended in 100 μl of nuclease-free water. Vector genome copies were quantified using the QX200 ddPCR system in ddPCR supermix for probes (no deoxyuridine triphosphate; Bio-Rad—no. 1863025) with primers/probes targeting eGFP and the housekeeping gene glyceraldehyde 3-phosphate dehydrogenase (GAPDH) (Hoerndli et al., 2004) (either Eurofins or Integrated DNA Technologies; Supplementary Table 1) following the manufacturer's instructions. The results were analyzed using QuantaSoft Analysis Pro Software (V1.0.596—Bio-Rad).

### 2.6 Quantification of mRNA expression by reverse transcriptase-ddPCR

Cells were harvested with TRIzol™ Reagent (Ambion—no. 15596018), and RNA was extracted following the manufacturer's instruction for RNA isolation. RNA pellets from TRIzol™ extracts were resuspended in DEPC Water (Ambion—no. AM9906) and were treated with 0.1 U/μl of DNase I (Ambion—no. AM2222) in 20 μl of DNase I Buffer (Ambion—no. 8170G) for 1 h at 37°C. DNase was inactivated by adding 25 mM of EDTA and heating to 65°C for 15 min. cDNA was generated using the iScript™ cDNA Synthesis Kit (Bio-Rad—no. 1708891) following the manufacturer's protocol. Nucleic acid expression profiles were quantified using the QX200 ddPCR system in ddPCR supermix for probes (no deoxyuridine triphosphate) with primers/probes targeting pDyn and GAPDH (Hoerndli et al., 2004) (either Eurofins or Integrated DNA Technologies; Supplementary Table 1), following the manufacturer's instructions. The results were analyzed with QuantaSoft Analysis Pro Software (V1.0.596—Bio-Rad).

### 2.7 Analysis of mature dynorphin B by enzyme-linked immunosorbent assay

For quantification of processed Dynorphin B (DynB), the medium and cell extracts from differentiated and undifferentiated SH-SY5Y were used and analyzed using enzyme-linked immunosorbent assay (ELISA).

For the quantification of intracellular Dynorphin, cells were harvested in 1X ELISA Lysis Buffer (137 mM NaCl; 20mM Tris-HCl, pH 7.5; 1% Triton X-100; 10% Glycerol) supplemented with 1X Halt Protease and Phosphatase Inhibitor Cocktail (Thermo Scientific—no. 78440). The cells were incubated on ice for 15 min, followed by centrifugation at 14,000 × *g* for 15 min at 4°C. The supernatant was diluted 1:5 in DPBS in a fresh tube. Samples were acidified to pH 2.6 by adding 1 M HCl and incubated for 15 min at RT. The samples were neutralized to pH 7.6 with 1 M NaOH. The medium was used without further treatment.

DynB from the cell extracts or the medium was quantified using the DynB ELISA Kit (BMA Biomedicals—no. S-1429), following the manufacturer's protocol. ELISA plates were read on a Tecan Infinite F50 Plus plate reader (Tecan). The results were analyzed using Magellan for F50 Software (Tecan—Version 7.2).

### 2.8 Graphical representation and statistical analysis

Graphical representation and statistical analysis of the data and figures were performed using Prism V10.0.0 GraphPad software.

## 3 Results

### 3.1 AAV MOI-dependent eGFP expression in SH-SY5Y cells

**Figure 1:**
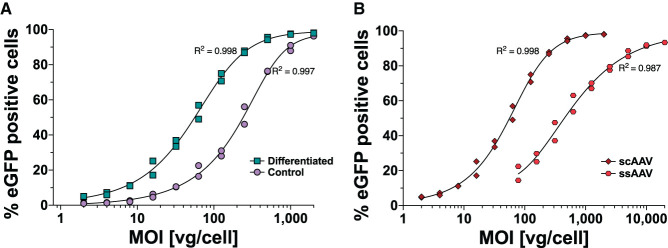
MOI-dependent eGFP expression profile in SH-SY5Y cells following AAV1-eGFP transduction measured by flow cytometry. **(A)** The expression profile of differentiated (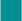 RA + TPA for 5 days) and undifferentiated (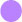 Control) SH-SY5Y cells transduced with scAAV-eGFP and harvested after 4 days for flow cytometry analysis is depicted. **(B)** GFP expression profiles following scAAV-eGFP (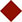 scAAV) and ssAAV-eGFP (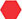 ssAAV) transduction in differentiated SH-SY5Y and harvested after 6 days are shown. Datapoints represent two independent experiments with five technical repeats each.

The differentiation of neuroblastoma cells is time-consuming and prone to well-to-well cell heterogenicity leading to intra-assay variability, thereby disturbing the quantitative analysis of AAV vector batch-to-batch variations. Therefore, we first investigated whether differentiation was necessary for efficient AAV transduction. SH-SY5Y was treated for 5 days before AAV transduction either with RA + TPA (in 0.2% DMSO) for differentiation or with 0.2% DMSO for undifferentiated controls. The cells were assessed using microscopy for morphological changes as described in previous studies (Kovalevich and Langford, 2013; Kovalevich et al., 2021) (Supplementary Figure 1). The cells were transduced with a scAAV-eGFP at a starting MOI of 2,000 followed by two-fold dilution steps down to MOI of 2. The cells were harvested 96 h after infection and analyzed for eGFP expression using flow cytometry (Figure 1A). Differentiated cells reached >95% eGFP-positive cells at MOI 500, while undifferentiated cells required four times more vector genomes per cell to yield equivalent expression. We investigated whether the difference between eGFP expressions in undifferentiated vs. differentiated SH-SY5Y cells reflected differences in AAV uptake. For this, we repeated the infection experiment using three biological replicates, harvested the cells 24 h after infection, washed the cells to remove any residual AAV, and quantified the transduced vector genome copies per cell. We found no difference in AAV uptake between differentiated and undifferentiated cells (Table 1). This suggested that the susceptibility of SH-SY5Y cells for AAV is not changed during the differentiation process.

**Table 1:** Vector genome copies per cell in differentiated and undifferentiated SH-SY5Y.

**MOI**	**Differentiated**		**Undifferentiated**	
	**MEAN**	**STD**	**MEAN**	**STD**
2,000	6.15	2.82	4.81	2.60
1,000	7.49	4.62	5.73	4.86
500	4.92	3.33	3.13	1.76
250	5.47	1.08	2.23	2.23
125	3.80	0.92	2.10	0.87
63	2.28	0.45	0.84	0.41
31	0.84	0.72	0.60	0.53
16	0.39	0.09	0.33	0.29
8	0.64	0.74	0.59	0.81
4	0.36	0.35	0.13	0.08
2	0.24	0.29	0.07	0.04
0	0.00	0.00	0.00	0.00

MOI, multiplicity of infection; STD, standard deviation.

To overcome the need for ssAAV to complete second-strand DNA synthesis before mRNA synthesis, McCarty et al. (2001) and McCarty et al. (2003) developed scAAV vectors by mutating one of the terminal repeats, resulting in a truncated terminal resolution site. This leads to linking the positive and negative DNA strand sequences as a single molecule packaged in single AAV capsids. Moreover, an increase in transduction efficiency of scAAV over ssAAV has been shown in several organs such as the liver, central nervous system (McCarty et al., 2003), eyes (Gruenert et al., 2016; Wang et al., 2017), and a number of commonly used cell lines (Wang et al., 2003). McCarty et al. (2001, 2003) demonstrated that for 1 scAAV virion, 10 ssAAV are required to achieve equal transduction efficiency. We investigated the difference between scAAV-eGFP and ssAAV-eGFP expression in differentiated SH-SY5Y cells. The WPRE element was introduced in single-strand AAV vectors as a stuffer and to optimize transgene expression. Differentiated SH-SY5Y cells were transduced with a scAAV-eGFP at a starting MOI of 2,000 down to an MOI of 2. ssAAV-eGFP transduction started at MOI 20,000 and diluted to an MOI of 78. The cells were analyzed for eGFP expression after 6 days using flow cytometry (Figure 1B). scAAV transduced cells displayed eGFP expression at 10-fold lower MOIs compared to ssAAV.

### 3.2 mRNA expression following AAV-pDyn transduction

**Figure 2:**
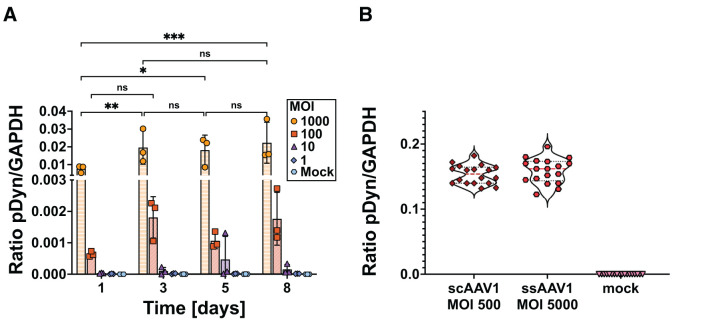
mRNA expression kinetics of preprodynorphin following ssAAV-pDyn/scAAV-pDyn transduction in differentiated SH-SY5Y cells. **(A)** The time course of pDyn mRNA expression from ssAAV-pDyn is depicted. Cells were transduced at decreasing MOIs: 1,000 (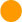), 100 (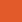), 10 (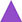), 1 (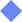), or mock infected (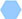). Cells were harvested at different time points (days 1, 3, 5, and 8 post-AAV transduction). mRNA expression levels were analyzed for pDyn by RT-ddPCR and normalized to the housekeeping gene GAPDH. Data show three biological replicates with mean ± SD. Analysis of variance with Tukey's multiple comparison test was performed for MOI 1,000 comparing the different time points (***P-value = 0.0003; **P-value = 0.0031; *P-value = 0.0110; ns, non-significant). **(B)** pDyn mRNA expression profiles following transduction of scAAV-pDyn (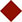) at an MOI of 500 and ssAAV-pDyn (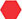) at an MOI of 5,000 are compared. Cells were harvested 96 h (±2 h) after infection and analyzed for pDyn mRNA expression as in **(A)**. Data are shown as nine technical replicates each from two biological replicates.

As a first step to validate AAV-pDyn transduction of SH-SY5Y cells, we analyzed mRNA expression with a ssAAV-pDyn at four different MOIs (1,000, 100, 10, and 1) and harvested cells at four different time points post-transduction (days 1, 3, 5, and 8). As shown in Figure 2A, at MOI 1,000, mRNA expression increased significantly compared to days 1 to 3, 5, and 8. Between days 3, 5, and 8, we saw no significant differences in mRNA expression, suggesting that a plateau has been reached between days 3 and 8. For MOI 100, a statistically non-significant 2.8-fold increase in mRNA expression was seen between days 1 and 3. At lower MOIs, hardly any AAV transduction can be expected and RNA levels were near to the lower detection limit. Indeed, we only found a significant MOI-dependent RNA increase between MOI 1,000 and 100 (Supplementary Figure 2).

To confirm the difference between ssAAV and scAAV in eGFP expression, scAAV and ssAAV variants of AAV-pDyn were applied at MOI of 500 and 5,000, respectively. In line with the results obtained for eGFP, both conditions yielded comparable levels of mRNA expression (Figure 2B). This confirms previous data obtained in liver cells, corneal endothelial cells, and muscle cells (Ding et al., 2003; McCarty et al., 2003; Gruenert et al., 2016; Collaud et al., 2019).

### 3.3 Dynorphin maturation process in SH-SY5Y measured by dynorphin B ELISA

**Figure 3:**
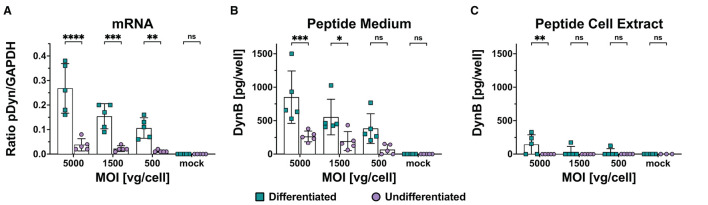
mRNA and mature Dynorphin B expression in differentiated and undifferentiated SH-SY5Y following scAAV-pDyn transduction. SH-SY5Y cells were transduced with scAAV-pDyn at three different MOIs (5,000, 1,500, and 500) and harvested 96 h post-transduction. pDyn mRNA expression **(A)** and Dyn B levels in the medium **(B)** and in the cell extract **(C)** were analyzed in parallel from each infection well (*n* = 5) of differentiated and undifferentiated SH-SY5Y cells. **(A)** mRNA expression of pDyn was normalized to GAPDH in differentiated cells (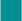) and undifferentiated cells (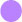). **(B)** Amount of DynB was quantified per well in the medium of differentiated cells (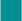) and undifferentiated cells (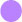). **(C)** Amount of DynB was quantified per well in the cell extract of differentiated cells (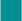) and undifferentiated cells (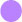). Data show five biological replicates with mean ± SD (****P-value ≤ 0.0001; ***P-value ≤ 0.001; **P-value ≤ 0.01; *P-value ≤ 0.05; ns—non-significant following a two-way ANOVA).

The maturation of biologically active neuropeptides from the precursor is a crucial issue when predicting the *in vivo* suitability of the vector. Differentiating between precursor and mature peptide can be achieved with antibodies detecting the free end of the peptide but showing no affinity to the peptide within the precursor. The DynB ELISA kit S-1429 (BMA, Augst, Switzerland) uses an antibody with minor cross-reactivity to big dynorphin but no affinity to other components of prodynorphin. We transduced differentiated and undifferentiated cells with scAAV-pDyn at MOIs of 5,000, 1,500, and 500. After 96 h, the supernatant was harvested for analysis. The cell pellets were split for parallel analysis of dynorphin peptide and mRNA. We observed a dose-dependent increase of pDyn mRNA expression and pDyn peptide in the medium, which was significantly higher in differentiated than in undifferentiated cells (Figures 3A, B, Supplementary Table 2). In the cell extracts, DynB was barely detectable in differentiated cells and below the limits of detection in undifferentiated cell extract (Figure 3C). No dose-dependency was observed (Supplementary Table 2). This suggests that DynB was liberated into the medium. In undifferentiated cells, the mRNA expression was already marginal and did not lead to quantifiable DynB levels (Figure 3). Despite utmost care to treat cells in a reproducible manner, we observed high variability between biological replicates with coefficients of variation above 45% for DynB detected in the medium and above 30% for RNA expression (Supplementary Table 3).

## 4 Discussion

In this study, we have explored the suitability of SH-SY5Y as a model for studying neuropeptide-expressing AAV1 vectors. We have shown that differentiating SH-SY5Y for 5 days with RA and TPA led to an increase in transduction efficiency as well as mRNA and protein expression. Additionally, we have demonstrated that SH-SY5Y was able to process precursor neuropeptides into mature neuropeptides.

The SH-SY5Y cell line represents one of the few human neuroblastoma cell lines that can be differentiated into mature neurons (Ross et al., 1983; Preis et al., 1988; Nishida et al., 2008) and were therefore extensively used as CNS disease models in Parkinson's or Alzheimer's disease (Xie et al., 2010; Kovalevich and Langford, 2013; Xicoy et al., 2017). SH-SY5Y cells were also used in combination with DNA delivery by AAV vectors (Garrity-Moses et al., 2005; Dudek et al., 2010; You et al., 2012; Kermer et al., 2015; Lewis et al., 2015; Osborne et al., 2018; Patricio et al., 2018).

AAVs are single-stranded DNA viruses, which traffic to the cell nucleus, where the ssDNA genome is unpacked. Complementary-strand DNA synthesis is required before transcription can be initiated. This has been described as a major bottleneck of AAV transduction (Ferrari et al., 1996; Nakai et al., 2000) and was overcome by the mutation of one terminal resolution site to create a scAAV genome, which self-anneals upon genome release and thus accelerates transgene expression (McCarty et al., 2001, 2003). Several studies have shown the differences between scAAV and ssAAV expression in various tissue types and cell models (Loeb et al., 1999; Paterna et al., 2000; Gruenert et al., 2016; Wang et al., 2017). We show here for the first time a 10-fold difference between scAAV and ssAAV transduced gene expression upon transduction of differentiated human neuroblastoma cells.

Despite the excellent permissiveness of SH-SY5Y cells for AAV1, an important aspect is the ability of the cell line to express the therapeutic transgene and reproduce its biological mechanism of action. In the case of neuropeptides, this requires the presence of specific enzymes. In neurons, the precursor pDyn is sorted into large dense core vesicles where it is cleaved by prohormone convertase 1, 2 and carboxypeptidase E (Schwarzer, 2009). Without this processing, the peptides are not active and will not bind to their respective receptors. Transcription data of SH-SY5Y cells (differentiated and undifferentiated) reveal that SH-SY5Y cells do not express these prohormone convertases though they do express furin and other endopeptidases (Harenza et al., 2017; Rodriguez et al., 2019). These could potentially take over, recognize the cleavage sites, and process the precursor protein into the individual mature peptides though at a potentially lower and less accurate rate. Despite the fact that the required convertases were not expressed, mature DynB could be measured in the medium of differentiated SH-SY5Y, while DynB is close to the detection limit in the respective cell extracts. This suggests that prodynorphin is sorted into and processed in constitutive vesicles in the absence of large dense-core vesicles. We can exclude that endogenous background dynorphin is released and accounts for this effect as no DynB was present in the medium of mock control. In line with this, transcriptome data from different labs showed that SH-SY5Y cells do not express endogenous dynorphin (Harenza et al., 2017; Rodriguez et al., 2019).

In conclusion, our study shows for the first time that differentiation of SH-SY5Y cells with RA and TPA not only increases the transducibility and mRNA production of rAAV vector-delivered neuropeptide precursors but also can enhance the production and secretion of mature neuropeptides. This makes them a highly interesting tool for *in vitro* validation of neuropeptide-expressing vectors, though further studies are needed to identify whether other neuropeptides show the same requirement to be processed into their mature form.

## Data availability statement

The original contributions presented in the study are included in the article/Supplementary material, further inquiries can be directed to the corresponding authors.

## Ethics statement

Ethical approval was not required for the studies on humans in accordance with the local legislation and institutional requirements because only commercially available established cell lines were used.

## Author contributions

JZ: Conceptualization, Formal analysis, Investigation, Visualization, Writing—original draft. DH: Conceptualization, Formal analysis, Investigation, Writing—review & editing. AS: Conceptualization, Formal analysis, Investigation, Writing—review & editing. SL-P: Investigation, Writing—review & editing. E-MH: Investigation, Writing—review & editing. CS: Conceptualization, Formal analysis, Supervision, Writing—review & editing. RH: Conceptualization, Formal analysis, Funding acquisition, Supervision, Writing—review & editing.
