# Supplementary material for: Evaluation of the SH-SY5Y cell line as an in vitro model for potency testing of a neuropeptide-expressing AAV vector
Source: Front Mol Neurosci. 2023 Nov 30;16:1280556. doi: 10.3389/fnmol.2023.1280556 (PMC10720649; doi:10.3389/fnmol.2023.1280556)
Supplement: Supplementary file 1 [file Data_Sheet_1.PDF]

Supplementary Table 1: Primers and Probes used in this study

PRIMERS	5' – 3' SEQUENCE	MODIFICATIONS
**eGFP Forward**	CCACTACCTGAGCACCCAGTC	
**eGFP Reverse**	GGTCACGAACTCCAGCAGGAC	
**eGFP Probe**	AGCAAAGACCCCAACGAGAAGCGC	[5']6-FAM/ZEN/[3']IABkFQ
**PDYN Forward**	ATGTAGCCTGTGCGCCGTGAAAAC	
**PDYN Reverse**	GTCCTCTTTGTCGTTCAGGCCCAG	
**PDYN Probe**	TCAGGCCGCTCTGCTGCCTAGCG	[5']6-FAM-[3']BHQ1
**GAPDH Forward**	ATTCCACCCATGGCAAATTC	
**GAPDH Reverse**	GGGATTTCCATTGATGACAAGC	
**GAPDH Probe**	CACCGTCAAGGCTGAGAACGGGA	[5']HEX/ZEN/[3']IABkFQ
**mouse GAPDH Forward**	CCTCAACTACATGGTCTACATGTTCC	
**mouse GAPDH Reverse**	CTTCCCATTCTCGGCCTTG	
**mouse GAPDH Probe**	CTCCACTCACGGCAAATTCAACG	[5']HEX/ZEN/[3']IABkFQ

BHQ1 – Black Hole Quencher 1, eGFP – enhanced Green Fluorescent Protein, FAM – fluorescein, GAPDH - Glyceraldehyde 3-phosphate dehydrogenase, HEX - hexachloro fluorescein, IBFQ – Iowa Black FQ, pDyn - Prodynorphin

Supplementary Table 2: Analysis of Variance followed by Sidak's multiple comparison test of dose-dependent increase in differentiated and undifferentiated SH-SY5Y following AAV-pDyn transduction

Sample	Mean Difference	95% CI to difference	Adjusted p-value
Differentiated RNA			
5000 vs 1500	0,114	0,013 to 0,215	0,025
1500 vs. 500	0,048	-0,053 to 0,149	0,535
500 vs. Mock	0,106	0,005 to 0,207	0,039
Undifferentiated RNA			
5000 vs 1500	0,016	-0,007 to 0,039	0,234
1500 vs 500	0,010	-0,013 to 0,033	0,608
500 vs Mock	0,012	-0,011 to 0,035	0,464
Differentiated Peptide Medium			
5000 vs 1500	297,5	-143,7 to 738,7	0,249
1500 vs. 500	169,5	-271,7 to 610,7	0,687
500 vs Mock	383,5	-57,84 to 824,5	0,099
Undifferentiated Peptide Medium			
5000 vs 1500	70,02	-80,7 to 220,7	0,549
1500 vs. 500	126,6	-24,0 to 277,3	0,114
500 vs Mock	67,94	-82,7 to 218,6	0,573
Differentiated Peptide Cell Extract			
5000 vs 1500	109,1	-36,9 to 255,1	0,179
1500 vs. 500	9,64	-136,4 to 155,6	0,997
500 vs Mock	24,82	-121,2 to 170,8	0,959
Undifferentiated Peptide Cell Extract			
5000 vs 1500	NA	NA	NA
1500 vs. 500	NA	NA	NA
500 vs Mock	NA	NA	NA

NA – not applicable as means and standard deviation was equal to zero for Dynorphin Peptide measured in the cell extract of undifferentiated SH-SY5Y

Supplementary Table 3: Coefficient of Variation of Figure 3 values

Sample	**Average Value**	**STD**	**%CV**
Differentiated RNA			
5000	2.68E-01	1.01E-01	37.54 %
1500	1.54E-01	5.13E-02	33.30 %
500	1.06E-01	4.16E-02	39.24 %
Mock	0.00E+00	0.00E+00	
Undifferentiated RNA			
5000	3.80E-02	2.49E-02	65.53 %
1500	2.20E-02	1.10E-02	49.79 %
500	1.20E-02	4.47E-03	37.27 %
Mock	0.00E+00	0.00E+00	
Differentiated Peptide Medium			
5000	8.50E+02	3.92E+02	46.11 %
1500	5.53E+02	2.66E+02	48.06 %
500	3.83E+02	2.23E+02	58.20 %
Mock	0.00E+00	0.00E+00	
Undifferentiated Peptide Medium			
5000	1.44E+02	1.45E+02	100.95 %
1500	3.45E+01	7.71E+01	223.61 %
500	2.48E+01	5.55E+01	223.61 %
Mock	0.00E+00	0.00E+00	
Differentiated Peptide Cell Extract			
5000	2.65E+02	7.96E+01	30.07 %
1500	1.95E+02	1.42E+02	72.98 %
500	6.79E+01	7.40E+01	108.93 %
Mock	0.00E+00	0.00E+00	
Undifferentiated Peptide Cell Extract			
5000	0.00E+00	0.00E+00	
1500	0.00E+00	0.00E+00	
500	0.00E+00	0.00E+00	
Mock	0.00E+00	0.00E+00	

STD – Standard Deviation, %CV – Coefficient of Variation in percentage
