# Supplementary material for: Evaluation of the SH-SY5Y cell line as an in vitro model for potency testing of a neuropeptide-expressing AAV vector
Source: Front Mol Neurosci. 2023 Nov 30;16:1280556. doi: 10.3389/fnmol.2023.1280556 (PMC10720649; doi:10.3389/fnmol.2023.1280556)
Supplement: Supplementary file 2 [file Data_Sheet_2.PDF]

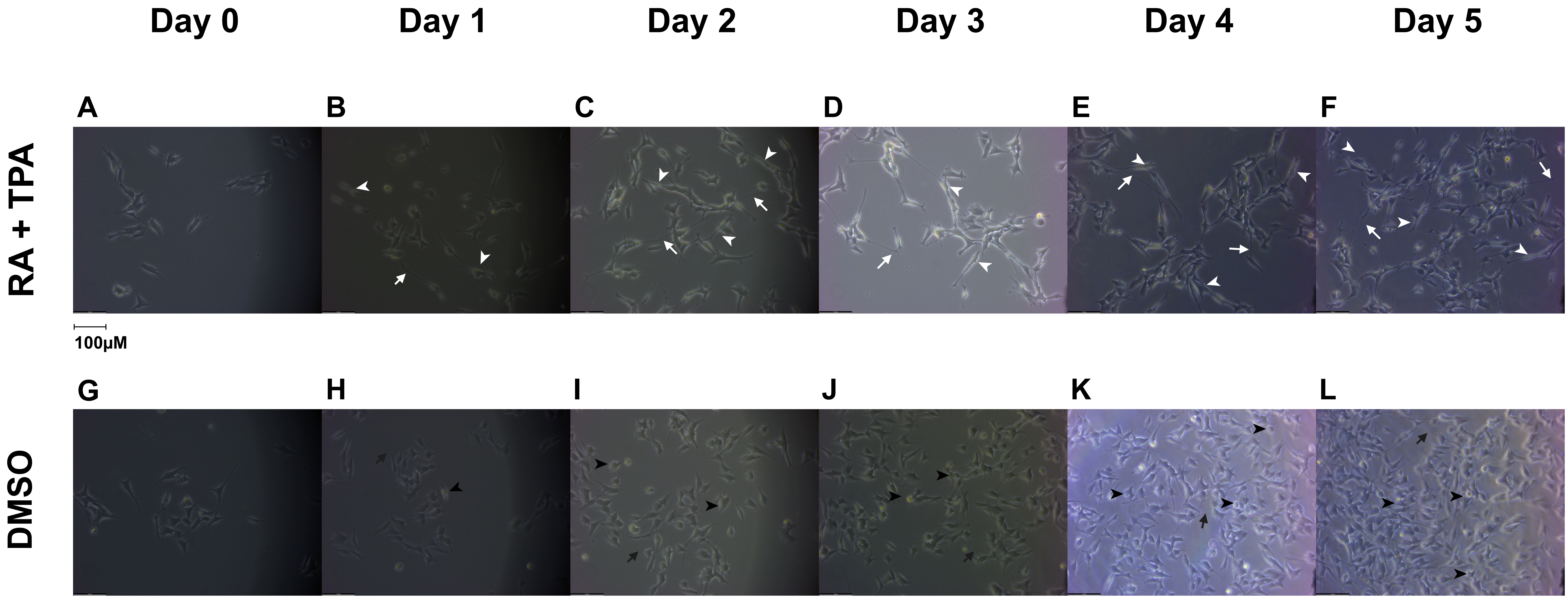

Supplementary Figure 1: Differentiation process of SH-SY5Y during treatment with RA and TPA compared to DMSO control for 5 days

Microscopy images of SH-SY5Y of cells during the differentiation process over days, either treated with RA + TPA (upper panel, A-F) or with DMSO (lower panel, G-L). Cells were assessed prior to addition of chemicals (Day 0 - A, G), and at days 1 (B, H), 2 (C,I), 3 (D, J), 4 (E, K) and 5 (F, L) after differentiation began. Cells were assessed morphologically for body elongation (white arrow head) and neurite growth (white arrow) in the RA + TPA group compared to round cells (black arrow head) and short neurite growth (black arrows) in the DMSO treated group. Elongated bodies with long neurite and processes are distinguished characteristics of differentiated SH-SY5Y compared to undifferentiated cells. Additionally, a decrease in proliferation rate has also been described (Kovalevich and Langford, 2013; Kovalevich, Santerre and Langford, 2021).

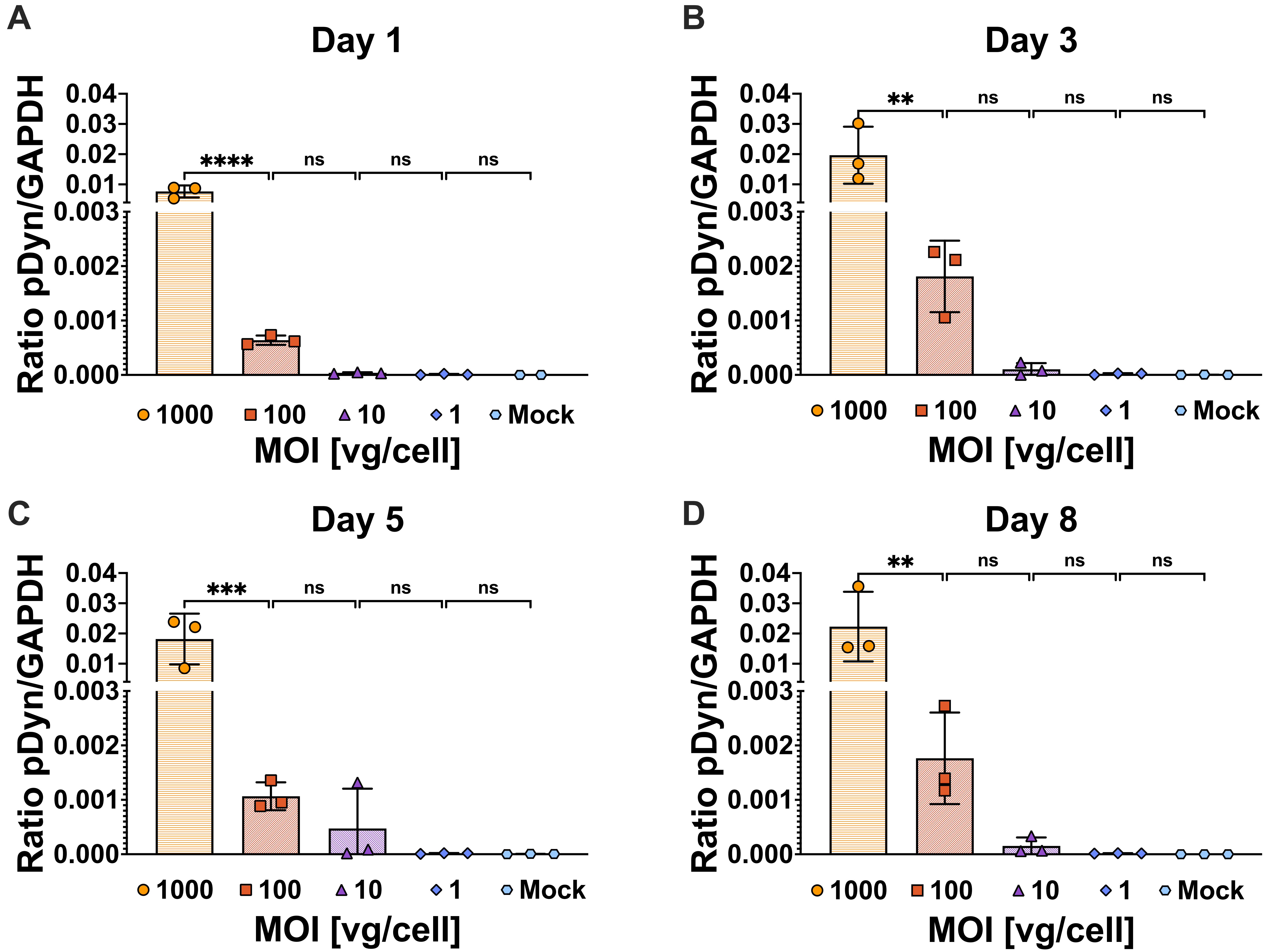

<u>Supplementary Figure 2:</u> Dose-dependency of ssAAV-pDyn mRNA Kinetic

Graphical representation of the statistical analysis of dose dependent mRNA expression from time kinetic following ssAAV-pDyn infection for each time point. Cells were transduced at decreasing MOIs: 1000 (●), 100(■), 10(▲), 1(◆), or mock infected (⬢). Cells were harvested at different time points (day 1 (**A**), 3 (**B**), 5 (**C**) and 8 (**D**) post-AAV transduction). Analysis of Variance followed by a Sidak's multiple comparison test was performed (**** - p-value < 0.0001 (A); ** - p-value = 0.0017 (B), *** - p-value = 0.0010 (C), ** - p-value = 0.0026 (D), ns - not significant).
